# Does awareness of condition help people with mild-to-moderate dementia to live well? Findings from the IDEAL programme

**DOI:** 10.1186/s12877-021-02468-4

**Published:** 2021-09-25

**Authors:** Catherine M. Alexander, Anthony Martyr, Laura D. Gamble, Sharon A. Savage, Catherine Quinn, Robin G. Morris, Rachel Collins, Linda Clare, in collaboration with the IDEAL programme research team

**Affiliations:** 1 REACH: The Centre for Research in Ageing and Cognitive Health, College of Medicine and Health, University of Exeter Medical School, University of Exeter, South Cloisters, St Luke’s Campus, Exeter, EX1 2LU UK; 2 Population Health Sciences Institute, Newcastle University, Newcastle upon Tyne, UK; 3 School of Psychology, The University of Newcastle, Australia, Newcastle, Australia; 4 Centre for Applied Dementia Studies, University of Bradford, Bradford, UK; 5 Department of Psychology, King’s College London, Institute of Psychiatry, Psychology and Neuroscience, London, UK; 6 National Institute for Health Research Applied Research Collaboration South-West Peninsula, Exeter, UK

**Keywords:** Anosognosia, Insight, Diagnosis, Disclosure, Quality of life, Well-being, Life satisfaction, Caregiver stress

## Abstract

**Background:**

People living with dementia vary in awareness of their abilities. We explored awareness of the condition and diagnosis in people with mild-to-moderate dementia, and how this relates to quality of life, well-being, life satisfaction, and caregiver stress.

**Methods:**

This study was a cross-sectional exploratory analysis of data from the IDEAL cohort, which recruited people with dementia living at home and available caregivers from 29 research sites in Great Britain. Our study included 917 people with mild-to-moderate dementia and 755 carers. Low and high awareness groups were derived from self-reported responses to a dementia representation measure. Logistic regression was used to explore predictors of awareness of condition and diagnosis using demographic, cognitive, functional and psychological measures, and the relationship with quality of life, well-being and life satisfaction (‘living well’), and caregiver stress.

**Results:**

There were 83 people with low awareness of their condition. The remaining 834 people showed some awareness and 103 of these had high awareness of their condition and diagnosis. Psychosocial factors were stronger predictors of awareness than cognitive and functional ability. Those with higher awareness reported lower mood, and lower scores on indices of living well as well as lower optimism, self-efficacy and self-esteem. Low awareness was more likely in those aged 80y and above, and living in more socially deprived areas. No relationship was seen between caregiver stress and awareness.

**Conclusions:**

Awareness of the condition and diagnosis varies in people with mild-to-moderate dementia and is relevant to the capability to live well. Awareness should be considered in person-centered clinical care.

**Supplementary Information:**

The online version contains supplementary material available at 10.1186/s12877-021-02468-4.

## Background

Dementia is an acquired neurocognitive disorder [1] with progressive disturbance of thinking, behavior and ability to perform everyday activities. The worldwide prevalence is approximately 50 million, estimated to treble by 2050 [2]. Care provision for this increasingly large group of people is a global public health challenge.

Among people living with dementia some show more awareness of the condition and difficulties they encounter than others. Definitive estimates of the prevalence of impaired awareness are unhelpful as these vary widely according to how it is measured [3]. However, understanding an individual’s level of awareness is important for facilitating appropriate care. For example, people with dementia who have higher awareness are more prone to low mood or higher anxiety [4], and those with lower awareness may be at risk from undertaking dangerous activities or making unsafe choices [5].

There are differing conceptual models of awareness. Some models predominantly view lack of awareness as a symptom of brain disease and neurocognitive deficits that can be mapped radiologically [6]. This may be termed anosognosia, meaning lack of knowledge of a deficit, typically neurological. Alternatively, a lack of awareness of a medical condition or symptom, commonly psychiatric, may be categorized as a lack of insight, possibly reflecting psychological processes of denial, which may be seen as a symptom of that condition. Other models consider how awareness is influenced by social constructs and established psychological responses to a given situation [7]. Cognitive models have attempted to explain how awareness is processed [8] and a combined approach proposes a model that regards biological, psychological and social influences [7, 9]. This broad biopsychosocial model incorporates the above concepts and defines awareness as ‘a reasonable or realistic perception or appraisal of a given aspect of one’s situation, functioning or performance, or of the resulting implications, which may be expressed explicitly or implicitly’ (7; p936). This model also allows for possible changes in expressed awareness enabled by the psychological and social processing of experiences and emotions [10]. This may reflect reaction to and/or adjustment to difficult situations caused by an unwelcome diagnosis [11], as seen in other progressive diseases.

Reviews note the contribution of different conceptual approaches and methodologies to the inconsistency in findings about factors associated with awareness [3, 12, 13]. These inconsistencies may have been compounded by the numerous methods used and the lack of a gold standard measure of awareness [14]. Poorer cognitive or functional abilities have sometimes been associated with lower awareness [15, 16] but overall conclusions are unclear [3]. For any given stage of dementia there remains a spectrum of awareness [13] suggesting that factors beyond cognitive impairment or disease progression can affect awareness.

Awareness can be categorized by the object of awareness, for example awareness of memory problems or of functional difficulties in everyday activities, ability to manage social and emotional situations or awareness of the condition of dementia and its implications. Distinguishing between the objects of awareness under scrutiny may help explain the diverse findings [17]. Studies which compare awareness across different objects may be more effective in identifying the important correlates [18–20]. Furthermore, standardization of the type of method used to measure awareness may aid comparison across objects [9].

Using this approach it appears that increasing age is associated with reduced awareness of memory ability [9], functional ability [9, 20, 21] and condition [20]. Severity of neuropsychiatric symptoms may also be associated with poorer awareness of memory, functional ability and socio-emotional function [9]. Low mood has frequently been associated with better awareness of memory or cognition [9, 18–20] and with more awareness of functional ability [9, 20] and condition [19, 20]. Caregiver stress was higher where there was lower functional awareness [9, 18], and with lower memory awareness [9].

The dementia subtype most commonly studied is Alzheimer’s disease (AD); no clear pattern of association between awareness and subtype has been identified in these studies although elsewhere impaired general awareness has sometimes been associated with frontotemporal dementia [12]. Personality type has infrequently been studied in relation to awareness. Where included, there are no consistent correlations but having a stronger self-concept or having more self-certainty was associated with lower awareness of memory and socio-emotional functioning [9] and lower awareness of condition [20]. There is no clear association between sex or educational attainment with awareness across any object.

Most research in this area has looked at awareness of cognitive impairment, typically memory, and/or awareness of functional abilities. There has been less focus on awareness of the condition of dementia and few studies have looked directly at the implications of awareness of diagnosis and prognosis [22]. Looking further at this could contribute to a greater understanding of the experience of receiving a dementia diagnosis, and how best to offer post-diagnostic care.

To support people after a diagnosis of dementia an important current focus of policy is living well with the condition [23]. This can be assessed in a number of ways, including measuring quality of life (QoL), well-being and life satisfaction [24]. When investigating awareness and living well, most studies have used measures of QoL rather than well-being or life satisfaction [25] with mixed findings, complicated by different objects studied. When objects of awareness were differentiated [26], better functional and memory awareness were weakly associated with lower QoL with depression and self-concept as important mediating factors, but global awareness was not related to QoL.

Living well indices have been associated with the psychological resources of optimism, self-esteem and self-efficacy [27], perhaps acting through greater resilience to adversity. To our knowledge, awareness of dementia diagnosis and its association with living well and factors promoting resilience have not previously been explored and will be investigated here.

The aims of the study are to examine awareness of the condition of dementia in a large cohort of people with mild-to-moderate dementia [24, 28] and to answer the following questions:

1) What factors are associated with awareness of condition in people with dementia? Specifically, the study will investigate the following factors: age, sex, dementia subtype, time since diagnosis, education, level of social deprivation, cognitive ability, functional ability, whether depressed, personality traits and psychological resources (self-esteem, self-efficacy, and optimism).

2) In people with dementia, is awareness of condition associated with living well as indicated by quality of life, well-being and life-satisfaction measures?

3) Is awareness of condition associated with caregiver stress?

## Methods

### Design

This study presents an exploratory analysis of cross-sectional data from the Improving the experience of Dementia and Enhancing Active Life (IDEAL) cohort [24, 28].

### Study population

People with mild-to-moderate dementia of any type were recruited to the IDEAL cohort from 29 National Health Service sites across England, Scotland and Wales and the Join Dementia Research online platform, between July 2014 and August 2016. Join Dementia Research is a UK initiative to enable involvement in dementia research by people living with the condition and caregivers, by matching volunteers with suitable dementia research studies online. Entry requirements at baseline were a clinical diagnosis of dementia, a Mini-Mental State Examination [29] score of 15 or above and living in their own home. A caregiver was recruited alongside where possible. Exclusion criteria included inability to provide own consent to participate in the study, terminal disease, inability to speak English, and any possibility for home visits to pose a danger to researchers. For the first wave of data collection, participants were visited in their own home by researchers for structured interviews over 3 visits.

IDEAL was approved by the Wales Research Ethics Committee 5 (reference 13/WA/0405), and the Ethics Committee of the School of Psychology, Bangor University (reference 2014–11,684) and was registered with UKCRN, registration number 16593. This study reports baseline data from v4.5 of the dataset which comprised 1537 participants with dementia and 1277 caregivers*.*

### Measures used

#### Awareness

Responses from the self-report version of the Representations and Adjustment to Dementia Index (RADIX) were used as an indication of awareness of the condition of dementia. RADIX [30] was developed and validated to measure dementia representations by people with dementia. The initial nine questions act as a checklist to screen for a lack of awareness, by checking if the person acknowledges difficulties or changes that are typically associated with a dementia diagnosis (see Additional file 1: Supplementary Table 1). If no difficulties or changes are acknowledged then the rest of the measure eliciting understanding of these is not administered. For the respondents who show some acknowledgement, the measure continues with 14 more detailed questions about their understanding of the condition and its implications and consequences. These questions were developed through analysis of data from qualitative interviews in which people with mild-to-moderate dementia discussed their perceptions and experiences [31].

The screening checklist has been used here to indicate low awareness if none of the nine screening items were endorsed. For the rest of the cohort who endorsed at least one of the screening questions, the remaining RADIX measure was administered. Among this group, responses to four selected questions were used to identify a higher awareness group (see Table 1). Specifically, people with dementia who used a diagnostic label to describe their condition, who were able to give the medical diagnosis and attributed the condition to causes within the brain or a physical illness or disease that was going to worsen formed the high awareness group.

**Table 1:** RADIX questions used to create high awareness group

RADIX Item ***(Dementia representation component)***	Possible responses	Response indicating ‘high awareness’ ^**a**^
What do you call this difficulty or condition that you have? *(Identity)*	(How does the person refer to condition, e.g. dementia, memory problems, forgetfulness etc. Used as ‘identity label’ in further questions)	• Gives a diagnostic label indicating dementia.
Are you aware of a specific diagnosis? What does the doctor call it? *(Diagnostic identity)*	(Is a specific diagnosis given, including ‘dementia’ or specific dementia subtype)	• Acknowledges diagnosis and gives specific diagnosis.
What do you think caused or causes your [identity label]? *(Cause)*	• Ageing • Changes within the brain • Illness/disease/physical condition • Hereditary condition • Lifestyle/life events • Don’t know	• Changes within the brain • Illness/disease/physical condition
What do you think will happen to your [identity label] over time? *(Timeline)*	• Get better • Stay the same as it is now • Get worse	• It will get worse (agree or strongly agree)

^a^Considered high awareness of condition if gave ‘high awareness’ responses to all four questions

#### Living well

Three self-report measures assessing living well were completed by participants with dementia: the Quality of Life in Alzheimer’s Disease Scale (QoL-AD [32]) using the total score, the World Health Organization-Five Well-being Index percentage score [33] and the Satisfaction with Life Scale total score [34]. For each of these measures, a higher score indicates greater perceived capability to live well.

#### Other measures

##### Demographic information

Information was recorded for participants’ age, here grouped into categories (<65y, 65-69y, 70-74y, 75-79y, 80 + y), sex, time since diagnosis (<1y, 1-2y, 3 + y), education level (no qualifications, school leaving certificate at 16y or at 18y, University), relationship with caregiver if involved in study (spouse/partner or other), dementia subtype, and area-level deprivation. Area deprivation was derived from postcode information and national statistics available on government websites, from which quintiles were computed, with the first quintile representing the 20% most deprived areas [35] .

##### Tests of cognition

Addenbrooke’s Cognitive Examination III (ACE-III [36]) was administered to assess cognitive ability using the total score and subdomains of attention, memory, verbal fluency, language and visuospatial ability, with higher scores indicating greater ability.

##### Self-report measures completed by the person with dementia

Mood was assessed with the 10-item Geriatric Depression Scale (GDS-10 [37]), using a binary variable with a cut-off score of 4 or more indicating depressed mood [38]*.* Personality was profiled using the Mini-International Personality Item Pool [39]. This measure assesses the 5 traits of extraversion, agreeableness, conscientiousness, neuroticism and intellect/imagination (referred to as ‘openness’); higher scores indicate a higher level of that trait. Other psychological features were assessed with a measure of optimism (Life-Orientation Test-Revised [40]), self-esteem (Rosenberg Self-Esteem Scale [41]), and self-efficacy (Generalized Self-Efficacy Scale [42]); higher scores indicate higher level of that attribute, see Lamont et al. (2020) for a more detailed description.

##### Measures completed by the caregiver as informant

Functional ability was measured using a modified 11-item version of the informant-rated Functional Activities Questionnaire [21, 43]. This version includes an additional item concerning telephone use. Higher scores indicate poorer perceived function. Neuropsychiatric symptoms in people with dementia as reported by the caregiver were recorded using the Neuropsychiatric Inventory Questionnaire (NPI-Q) total symptom score, with higher scores indicating more symptoms [44, 45].

##### Self-report measure completed by the caregiver

Caregiver stress was assessed with the Relative Stress Scale [46]. This is a 15 item scale with each item rated 0 to 4; higher scores indicate more stress.

### Analyses

**Fig. 1:**
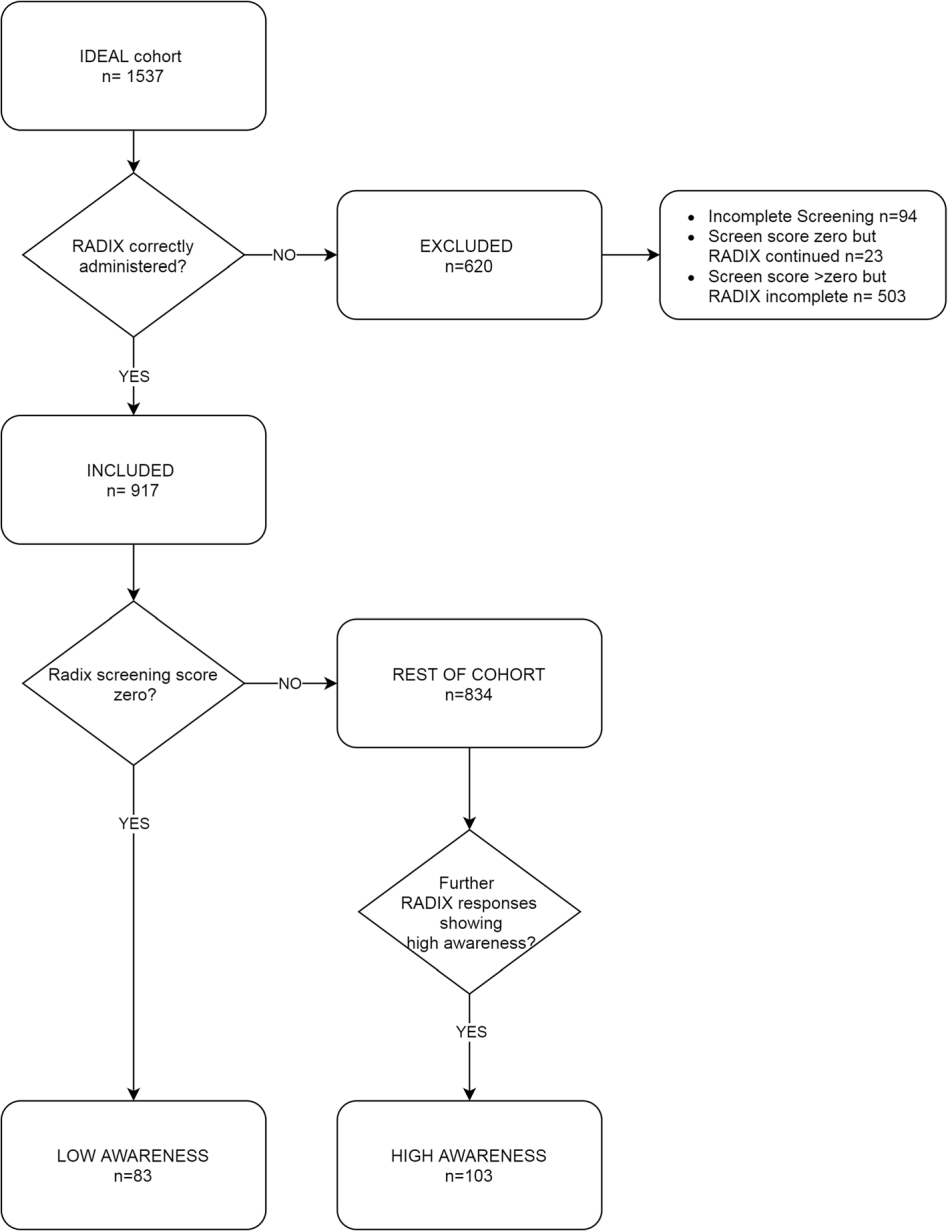
Flowchart showing formation of awareness groups

Participants were included if all nine RADIX screening questions were administered and, where indicated, subsequent full administration of the RADIX questionnaire was completed. Low and high awareness groups were derived as detailed above and illustrated in Fig. 1.

Using univariate logistic regression, an initial model showed the relationship between awareness group and demographic, cognitive and psychological variables, living well indices and caregiver stress. A second model adjusted for age group, sex and dementia subtype. A third model additionally adjusted for mood using the GDS-10 binary variable. In reporting the results, low awareness is discussed in relation to the comparison between the low awareness group and the rest of the cohort, and high awareness is illustrated by the comparison between the high awareness and low awareness groups. Results are interpreted with consideration of the odds ratios as effect sizes.

## Results

There were 917 people with dementia included in the analysis, of whom 755 had a participating caregiver. The majority of people with dementia were male (58.2%) and the largest diagnostic group was AD (55.4%). Ages ranged from 43y to 95y and the largest age group represented was aged 80y and above. The majority of caregivers were spouses or partners (68.7%) whilst 17.7% of participants had no caregiver involved in the study. No difference in demographic characteristics was seen in the excluded participants. Further information about study participants is reported in Table 2a-c.

**Table 2:** Description of participants

**a. Personal and demographic variables**				
	**Whole sample (*n*** **= 917)**	**Low awareness group (*n*** **= 83)**	**Rest of cohort including high awareness group** **(*n*** **= 834)**	**High awareness group (*n*** **= 103)**
	**N (%)**	**N (%)**	**N (%)**	**N (%)**
**Sex**				
Female	383 (41.8)	33 (39.8)	350 (42.0)	44 (42.7)
Male	534 (58.2)	50 (60.2)	484 (58.0)	59 (57.3)
**Age at baseline**				
< 65y	87 (9.5)	6 (7.2)	81 (9.7)	24 (23.3)
65-69y	120 (13.1)	11 (13.3)	109 (13.1)	16 (15.5)
70-74y	160 (17.4)	9 (10.8)	151 (18.1)	22 (21.4)
75-79y	216 (23.6)	12 (14.5)	204 (24.5)	23 (22.3)
80+	334 (36.4)	45 (54.2)	289 (34.7)	18 (17.5)
Mean (SD)	75.78 (8.46)	78.34 (9.14)	75.62 (8.35)	71.32 (9.08)
**Time since diagnosis**				
< 1y	477 (52.0)	39 (47.0)	438 (52.5)	42 (40.8)
1-2y	264 (28.8)	19 (22.9)	245 (29.4)	38 (36.9)
3y and above	108 (11.8)	11 (13.3)	97 (11.6)	18 (17.5)
Missing	68 (7.4)	14 (16.9)	54 (6.5)	5 (4.9)
**Education level**				
No qualification	253 (27.6)	22 (26.5)	231 (27.7)	29 (28.2)
School leaving certificate 16y	168 (18.3)	15 (18.1)	153 (18.3)	15 (14.6)
School leaving certificate 18y	304 (33.2)	26 (31.3)	278 (33.3)	35 (34.0)
University	173 (18.9)	14 (16.9)	159 (19.1)	24 (23.3)
Missing	19 (2.1)	6 (7.2)	13 (1.6)	0
**Dementia subtype**				
AD	508 (55.4)	47 (56.6)	461 (55.3)	53 (51.5)
VaD	105 (11.5)	12 (14.5)	93 (11.2)	16 (15.5)
Mixed	177 (19.3)	13 (15.7)	164 (19.7)	14 (13.6)
FTD	34 (3.7)	7 (8.4)	27 (3.2)	3 (2.9)
PDD	33 (3.6)	1 (1.2)	32 (3.8)	10 (9.7)
DLB	37 (4.0)	1 (1.2)	36 (4.3)	4 (3.9)
Other	23 (2.5)	2 (2.4)	21 (2.5)	3 (2.9)
**Deprivation quintile**				
Q1 (most deprived)	88 (9.6)	13 (15.7)	75 (9.0)	8 (7.8)
Q2	131 (14.3)	13 (15.7)	118 (14.1)	18 (17.5)
Q3	205 (22.4)	16 (19.3)	189 (22.7)	21 (20.4)
Q4	225 (24.5)	19 (22.9)	206 (24.7)	25 (24.3)
Q5 (least deprived)	268 (29.2)	22 (26.5)	246 (29.5)	31 (30.1)
**Caregiver relationship**				
No caregiver in study	162 (17.7)	16 (19.3)	146 (17.5)	21 (20.4)
Spouse/partner	630 (68.7)	56 (67.5)	574 (68.8)	72 (69.9)
Other	117 (12.8)	11 (13.3)	106 (12.7)	9 (8.7)
Missing	8 (0.9% of total, or 1.1% of those with caregiver)	–	8 (1% of total, or 1.2% of those with caregiver)	1 (1% of total, or 1.2% of those with caregiver)
**b. Cognitive and psychological variables**				
	**Whole sample (*n =*** **917)**	**Low awareness group (*n =*** **83)**	**Rest of cohort including high awareness group** **(*n =*** **834)**	**High awareness group (*n =*** **103)**
	**Mean (SD); n**	**Mean (SD); n**	**Mean (SD); n**	**Mean (SD); n**
**ACE-III total**	69.33 (13.07); 846	65.03 (13.21); 71	69.72 (12.99); 775	71.33 (13.12); 97
**ACE-III attention**	13.95 (2.96); 889	13.10 (3.24); 77	14.03 (2.92); 812	14.04 (3.01); 100
**ACE-III fluency**	6.77 (3.10); 894	6.26 (3.19); 77	6.82 (3.09); 817	6.93 (3.05); 101
**ACE-III language**	22.55 (3.49); 866	21.79 (3.67); 73	22.62 (3.46); 793	23.40 (3.11), 99
**ACE-III memory**	13.60 (5.31); 877	11.55 (5.02); 74	13.79 (5.30); 803	14.73 (5.34); 98
**ACE-III visuospatial**	12.45 (3.33); 882	12.41 (3.08); 74	12.45 (3.35); 808	12.45 (3.37); 100
**Personality trait**				
**Neuroticism**	10.12 (3.42); 882	8.10 (2.96); 81	10.33 (3.40); 801	10.94 (3.44); 100
**Conscientious**	13.61 (3.07); 882	14.85 (2.35); 81	13.48 (3.10); 801	13.14 (3.41); 99
**Openness**	12.87 (3.13); 874	13.23 (3.20); 79	12.84 (3.12); 795	12.68 (3.37); 99
**Agreeable**	15.81 (2.79); 886	15.43 (2.90); 81	15.85 (2.77); 805	16.02 (2.93); 100
**Extraversion**	11.77 (3.67); 886	12.69 (3.42); 81	11.68 (3.69); 805	10.69 (3.83); 99
**QoL-AD**	36.91 (5.97); 850	40.97 (5.02); 76	36.51 (5.91); 774	35.25 (5.91); 95
**WHO-5**	61.19 (20.84); 911	71.28 (19.20); 83	60.18 (20.74); 828	55.65 (21.30); 103
**SwLS**	26.20 (6.18); 905	29.02 (5.16); 82	25.91 (6.20); 823	23.22 (6.50); 103
**Optimism**	15.01 (3.58); 883	16.36 (3.55); 77	14.88 (3.56); 806	14.18 (3.99); 99
**Self-efficacy**	29.47 (5.51); 868	31.81 (5.77); 74	29.26 (5.43); 794	27.39 (5.97); 99
**Self-esteem**	29.50 (3.75); 851	31.36 (3.93); 72	29.33 (3.69); 779	28.93 (3.61); 98
	**N (%)**	**N (%)**	**N (%)**	**N (%)**
**GDS-10 group**				
Not Depressed	630 (68.7)	74 (89.2)	556 (66.7)	61 (59.2)
Depressed	267 (29.1)	7 (8.4)	260 (31.2)	40 (38.8)
Missing	20 (2.2)	2 (2.4)	18 (2.2)	2 (1.9)
**c. Caregiver-rated variables**				
	**Whole sample (*n =*** **917)**	**Low awareness group (*n =*** **83)**	**Rest of cohort including high awareness group** **(*n =*** **834)**	**High awareness group (*n =*** **103)**
	**Mean (SD); n**	**Mean (SD); n**	**Mean (SD); n**	**Mean (SD); n**
**FAQ-I**	17.46 (8.56); 697	20.69 (8.18); 62	17.15 (8.54); 635	16.85 (8.08); 74
**NPI-Q total**	3.52 (2.46); 717	3.34 (2.23); 64	3.53 (2.48); 653	3.60 (2.48): 77
**Caregiver RSS**	19.07 (9.98); 709	18.55 (8.71); 64	19.12 (10.10); 645	19.87 (8.72): 79

*AD* Alzheimer’s disease, *VaD* vascular dementia, *FTD* frontotemporal dementia, *PDD* Parkinson’s disease dementia, *DLB* dementia with Lewy bodies, *ACE-III* Addenbrooke’s Cognitive Examination III, *QoL-AD* Quality of Life in Alzheimer’s Disease, *SwLS* Satisfaction with Life Scale, *WHO-5* World Health Organization-Five Well-being Index, *GDS-10* Geriatric Depression Scale-10, *FAQ-I* Functional Activities Questionnaire-Informant rated, *NPI-Q* Neuropsychiatric Inventory Questionnaire, *RSS* Relative Stress Scale

There were 83 people identified as having low awareness (9.1%). From the remaining 834 who showed some awareness of their condition 103 people were identified as having high awareness (11.2%). Univariate logistic regression showed that the factors most strongly predictive of awareness group were age and mood (Additional file 2: Supplementary Table S2.1a- S2.3). People with depressed mood were more likely to be in the high awareness group, with 38.8% of the high awareness group categorized as having depressed mood, compared to 31.2% in the rest of the cohort and only 8.4% in the low awareness group. People with dementia aged 80y and above were more likely to be in the low awareness group whereas those under the age of 80y were more likely to have higher awareness, particularly those under 65y. Sex was not a predictor of awareness. Low numbers in the rare diagnostic subtypes meant that associations between dementia subtype and awareness were not clear. However, there was an over-representation of people with frontotemporal dementia in the low awareness group, and of people with Parkinson’s disease dementia and dementia with Lewy bodies in the high awareness group; see Additional file 4: Supplementary Fig. S1.

After adjusting for age group, sex and dementia subtype (Table 3) the significant likelihood of depressed mood predicting awareness group remained (Table 3c). Those who reported being more depressed were more likely to be in the high than the low awareness group, and less likely to be in the low awareness group compared to the rest of the cohort.

**Table 3:** Multivariate logistic regression awareness groups, adjusted for age group, sex, dementia subtype

Factors associated with awareness								
a. Demographic variables								
	**OR Low awareness (*n =*** **83) vs Rest of cohort (*n =*** **834)**				**OR High awareness (*n =*** **103) vs Low awareness (*n =*** **83)**			
	**OR**	**(95% CI)**	***p*-value**	**Missing cases (%)**	**OR**	**(95% CI)**	***p-*value**	**Missing cases (%)**
**Time since diagnosis**				68 (7.4)				19 (10.2)
< 1 yr	Reference group				Reference group			
1-2 yr	.92	(.51, 1.64)	.767		2.21	(.93, 5.25)	.074	
3 yr and above	1.39	(.67, 2.88)	.380		2.06	(.73, 5.81)	.173	
**Deprivation quintile**				0				0
Q1 (most deprived)	2.02	(.95, 4.30)	.068		.17	(.04, .75)	.019	
Q2	1.33	(.64, 2.76)	.444		.63	(.22, 1.80)	.387	
Q3	1.01	(.51, 2.00)	.973		.55	(.19, 1.55)	.257	
Q4	1.15	(.60, 2.20)	.686		.66	(.24, 1.77)	.405	
Q5 (least deprived)	Reference group				Reference group			
**Education**				19 (2.1)				6 (3.2)
No qualification	1.04	(.56, 1.93)	.899		1.32	(.53, 3.28)	.546	
School leaving certificate 16y	1.19	(.60, 2.36)	.611		.49	(.17, 1.39)	.179	
School leaving certificate 18y	Reference group				Reference group			
University	.99	(.49, 1.98)	.971		.93	(.34, 2.51)	.884	
b. Cognitive variables								
	**OR Low awareness (*n =*** **83) vs Rest of cohort (*n =*** **834)**				**OR High awareness (*n =*** **103) vs Low awareness (*n =*** **83)**			
	**OR**	**(95% CI)**	***p-*value**	**Missing cases (%)**	**OR**	**(95% CI)**	***p-*value**	**Missing cases (%)**
**ACE-III total**	.98	(.96, .99)	.007	71 (7.7)	1.04	(1.01, 1.07)	.016	18 (9.7)
**ACE-III attention**	.89	(.83, .97)	.005	28 (3.1)	1.15	(1.02, 1.29)	.022	9 (4.8)
**ACE-III fluency**	.94	(.87, 1.02)	.136	23 (2.5)	1.10	(.98, 1.24)	.106	8 (4.3)
**ACE-III language**	.95	(.89, 1.01)	.094	51 (5.6)	1.15	(1.03, 1.29)	.015	14 (7.5)
**ACE-III memory**	.93	(.88, .97)	.003	40 (4.4)	1.10	(1.01, 1.18)	.021	14 (7.5)
**ACE-III visuospatial**	.98	(.91, 1.06)	.642	35 (3.8)	1.06	(.95, 1.19)	.312	12 (6.5)
c. Psychological variables								
	**OR Low awareness (*n =*** **83) vs Rest of cohort (*n =*** **834)**				**OR High awareness (*n =*** **103) vs Low awareness (*n =*** **83)**			
	**OR**	**(95% CI)**	***p-*value**	**Missing cases (%)**	**OR**	**(95% CI)**	***p-*value**	**Missing cases (%)**
**GDS-10 group**				20 (2.2)				4 (2.2)
Depressed	.23	(.10, .51)	<.001		4.51	(1.70, 11.92)	.002	
Not depressed	Reference group				Reference group			
**Neuroticism**	.81	(.75, .88)	<.001	35 (3.8)	1.26	(1.11, 1.43)	<.001	5 (2.7)
**Openness**	1.03	(.96, 1.11)	.445	43 (4.7)	.97	(.87, 1.09)	.631	8 (4.3)
**Agreeableness**	.95	(.88, 1.04)	.259	31 (3.4)	1.12	(.99, 1.28)	.080	5 (2.7)
**Conscientious**	1.16	(1.07, 1.25)	.001	35 (3.8)	.86	(.76, .98)	.023	6 (3.2)
**Extraversion**	1.08	(1.01, 1.15)	.019	31 (3.4)	.86	(.78, .95)	.003	6 (3.2)
**Optimism**	1.12	(1.04, 1.20)	.002	34 (3.7)	.87	(.79, .96)	.007	10 (5.4)
**Self-efficacy**	1.09	(1.04, 1.15)	<.001	49 (5.3)	.87	(.81, .94)	<.001	13 (7.0)
**Self-esteem**	1.15	(1.08, 1.23)	<.001	66 (7.2)	.85	(.76, .95)	.004	16 (9.7)
d. Caregiver-rated variables								
	**OR Low awareness (*n =*** **67) vs Rest of cohort (*n =*** **688)**				**OR High awareness (*n*** **= 82) vs Low awareness (*n =*** **67)**			
	**OR**	**(95% CI)**	***p-*value**	**Missing cases (%)**	**OR**	**(95% CI)**	***p-*value**	**Missing cases (%)**
**FAQ-I**	1.05	(1.02, 1.09)	.003	58 (7.7)	.94	(.89, .99)	.011	13 (8.7)
**NPI-Q total symptoms**	.97	(.87, 1.08)	.537	38 (5.0)	1.01	(.85, 1.21)	.881	8 (5.4)
**Awareness and ability to live well**								
	**OR Low awareness (*n =*** **83) vs Rest of cohort (*n =*** **834)**				**OR High awareness (*n =*** **103) vs Low awareness (*n =*** **83)**			
	**OR**	**(95% CI)**	***p-*value**	**Missing cases (%)**	**OR**	**(95% CI)**	***p-*value**	**Missing cases (%)**
**QoL-AD**	1.17	(1.11, 1.23)	<.001	67 (7.3)	.83	(.77, .90)	<.001	15 (8.1)
**WHO-5**	1.03	(1.02, 1.04)	<.001	6 (0.7)	.97	(.95, .99)	.001	0
**SwLS**	1.11	(1.05, 1.16)	<.001	12 (1.3)	.84	(.78, .91)	<.001	1 (0.5)
**Awareness and caregiver stress**								
	**OR Low awareness (*n =*** **67) vs Rest of cohort (*n =*** **688)**				**OR High awareness (*n =*** **82) vs Low awareness (*n =*** **67)**			
	**OR**	**(95% CI)**	***p-*value**	**Missing cases (%)**	**OR**	**(95% CI)**	***p-*value**	**Missing cases (%)**
**Caregiver RSS**	.99	(.97, 1.02)	.674	46 (6.1)	1.01	(.96, 1.06)	.773	6 (4.0)

*OR* Odds ratio, *CI* Confidence interval, *AD* Alzheimer’s disease, *VaD* vascular dementia, *FTD* frontotemporal dementia, *PDD* Parkinson’s disease dementia, *DLB* dementia with Lewy bodies, *ACE-III* Addenbrooke’s Cognitive Examination III, *QoL-AD* Quality of Life in Alzheimer’s Disease, *SwLS* Satisfaction with Life Scale, *WHO-5* World Health Organization-Five Well-being Index, *GDS-10* Geriatric Depression Scale-10, *FAQ-I* Functional Activities Questionnaire-Informant rated, *NPI-Q* Neuropsychiatric Inventory Questionnaire, *RSS* Relative Stress Scale

A third model adjusting for GDS-10 group as well as age group, sex and dementia subtype (Additional file 3: Supplementary Table S3.1a-S3.3) corroborated findings for the other factors, with clearer links between awareness group and deprivation and personality traits as detailed below.

There were consistent findings across the 3 models showing no relationship with education level, NPI-Q total symptoms or the personality trait openness. Higher awareness was more likely with better cognitive scores on ACE-III total and all ACE-III subdomains apart from visuospatial ability. Lower awareness was more likely with lower cognitive scores and more impaired informant-rated functional ability in all three models. See Table 3a-d, Additional file 2: Supplementary Tables S2.1a-d and Additional file 3: Supplementary Tables S3.1a-d.

As indicated by higher scores for personality traits, conscientiousness was more likely with low awareness, and neuroticism with higher awareness in all models. Extraversion was more closely related to low awareness, whilst agreeableness was more likely with higher awareness in the fully adjusted model (Additional file 3: Supplementary Table S3.1c). Optimism, self-efficacy and self-esteem remained predictors of awareness when adjusted for depressed mood, with higher scores in the low awareness group. Scores for self-efficacy in particular were lower in the high awareness group (Additional file 3: Supplementary Table S3.1c). In the initial models, there was a trend for greater deprivation with low awareness (Table 3a and Additional file 2: Supplementary Table S2.1a). Clarified in the fully adjusted model the odds ratios suggest that people living in the least deprived areas are more likely to show higher awareness, and those in the most deprived areas more likely to show lower awareness (Additional file 3: Supplementary Table S3.1a).

Regarding living well, there was a consistent relationship in all three models between awareness group and self-rated scores for quality of life, well-being and life satisfaction, with higher scores suggesting greater perceived capability to live well in the low awareness group and lower scores in the high awareness group (Table 3, Additional file 2: Supplementary Table S2.2, Additional file 5: Supplementary Figs. S2a-S2c). This finding was not attenuated after adjusting for mood (Additional file 3: Supplementary Table S3.2). Overall, mean scores for caregiver stress were low for the whole sample and no relationship was found between caregiver stress and awareness group in any model.

## Discussion

In a large sample of people living with mild-to-moderate dementia, novel use of a validated measure of dementia representations [30] allowed exploration of the differences between people with low and high awareness of the condition of dementia. The study focused on factors that signal the experience of those living with dementia and are therefore important when considering care needs. A subgroup showing no acknowledgement of difficulties typical of dementia reported better scores on self-reported indices of living well and other indicators of psychological health such as self-reported optimism, self-esteem and self-efficacy. Among those who acknowledged some difficulties, a subgroup categorized by high awareness of their dementia diagnosis and a recognition of its causes and consequences had lower self-rated scores on living well indices and psychological resources. Caregivers of people in each of these groups were not distinguishable by levels of reported stress. Age, mood and psycho-social factors were stronger predictors of the awareness group than the level of cognitive or functional impairment.

Whilst most of the sample showed some awareness, nearly 10 % appeared to have no explicit awareness of their difficulties. In line with earlier studies investigating other objects of awareness [12], older age and lower cognitive and functional ability were associated with lower awareness of condition, which would be consistent with impairment due to neurodegenerative processes. However, compared to cognitive and functional variables, larger effects were seen for some psychosocial factors i.e. mood, personality traits, psychological resources and level of deprivation, confirming the relative importance of these factors when discussing awareness of condition. It is important to recognize that some people are implicitly aware of their difficulties even when this is not overtly expressed [47], which may govern the choices made or reactions to situations in everyday life [48]. A small proportion of the low awareness group gave self-ratings indicating low mood, so assumptions should not be made about the advantages of apparent low awareness, but being less aware of the condition of dementia may be beneficial for most in regard to mood.

Just over 10 % gave diagnostic labels such as ‘dementia’ or a specific subtype, with medically appropriate explanations for the cause and prognosis of their condition. This echoes other studies that found a minority of people with dementia use medical terms when referring to their diagnosis of dementia [49] and most use more general terms such as ‘memory problems’ or ‘forgetfulness’. Although this group were more likely to be younger, from less deprived areas and with fewer cognitive and informant-rated functional impairments, self-reports indicated lower psychological resources, lower mood and lower living well indices. Therefore, being diagnostically aware does not apparently help living well or psychological health.

Previous work has suggested that mood may be an important mediator between memory awareness and self-rated QoL [26, 50, 51]. One proposed mechanism is that depressed individuals are more likely to endorse difficulties due to negative bias and appear more aware [52]. Alternatively, being more aware of symptoms of dementia could understandably result in lower mood. After adjusting for depressed mood, findings indicate a persisting link between high awareness and lower scores on living well, suggesting this is due to appraisal of the situation rather than reflecting a negative bias due to low mood.

Nonetheless, there was wide variability for the living well indices and over half of the high awareness group were in the ‘not depressed’ group, suggesting that it may be possible to support people in gaining awareness without harming mood and ability to live well. There is no information available concerning how this group came to develop better awareness of their condition, whether it was a gradual process led by their own enquiry, or whether it was communicated at all or abruptly at an unwelcome time; practice in delivering the diagnosis is known to vary [53]. A phenomenological approach to awareness suggests there is a range of responses to receiving a diagnosis of dementia reflecting individual coping styles [11]. These range from ‘self-maintaining’ or normalizing experiences to ‘self-adjusting’ or facing up to the problem and adapting. Intervention studies suggest that for people who acknowledge at least some problems with memory, a safe place and time to discuss with others can lead to a gradual integration of the diagnosis [54], with possible improvement in QoL and self-esteem. The current cross-sectional study is consistent with other studies showing little association between awareness and the duration of the diagnosis [12], but gives little indication of where an individual may be in their personal process of adjusting to their dementia diagnosis. Longitudinal studies have indicated that awareness in other domains may worsen over time with disease progression [16] but a small minority show increased awareness [15, 55]. Investigating the longer-term consequences of awareness of diagnosis on living well, and whether awareness of diagnosis changes, would be clinically important for care provision. Identifying an individual’s position on the psychological and emotional processing that follows diagnosis could lead to focused interventions to support people through this adjustment.

Findings were consistent for all three measures of living well with largest effects seen with scores for QoL-AD, which is the most commonly used measure of QoL in dementia [25]. However, some other research disputes whether higher or lower awareness is associated with better QoL [25]. Differences may be explained by different objects of awareness investigated [26]. Awareness of cognitive or functional ability may be beneficial for outcomes of multicomponent interventions [56]. Goal-setting by people with dementia in a multi-disciplinary rehabilitation program was enabled by their awareness of motor and functional difficulties [57]. Likewise, some individuals with dementia who are aware of their diagnosis are involved in advocacy and have derived benefit in terms of group identity and making a difference [58]. Presumably there are situations where awareness of diagnosis might be more advantageous particularly if it aids involvement in decisions to start anti-dementia medication [22] or in accessing support and harnessing hope [53]*.*

These results suggest that awareness of dementia condition and diagnosis is not directly related to caregiver stress. Caregiver stress and burden have been associated with more severe neuropsychiatric symptoms as well as low awareness in other domains [3, 26]. Current findings suggest no association between number of neuropsychiatric symptoms and awareness, which may be reflected in the relatively low levels of caregiver stress. Alternatively, not acknowledging the condition of dementia may cause less stress to caregivers than having reduced awareness of everyday abilities which might result in higher risk and more conflict [5].

Consistent with earlier studies that investigated other objects, personality trait scores were weakly related to awareness of condition, particularly conscientiousness with lower awareness [59] and neuroticism and agreeableness with higher awareness [60]. In addition, low awareness was more likely with higher extraversion scores; elsewhere extraversion was a marginal factor regarding awareness of memory performance [20]. There is predictably some collinearity between self-reports for personality traits and psychological resources which may be reflected in the living well indices [27], but findings regarding awareness are salient. Personality could be interacting with awareness via personal coping style, or perhaps amenability to noticing and accepting feedback either from others or from failure experiences, allowing opportunity to update self-knowledge [8]. Notably though, a study using retrospective informant ratings of personality found no convincing association with awareness of condition [20].

Lower awareness of the condition of dementia was more likely for those living in the most deprived areas. As in other studies educational attainment did not appear to influence awareness [12]. A cross-cultural study of three global regions found more awareness of memory problems with higher socio-economic status in one cultural group [61]. In Asia, awareness of dementia varied in people with dementia and caregivers across seven locations, perhaps reflecting the degree of traditional culture and information available [62]. Awareness of diagnosis might be facilitated by access to information about dementia at home and from local dementia services, and opportunity to accept and adjust to the diagnosis. Low awareness could reflect lack of information, poor access to services, or unreadiness to acknowledge dementia perhaps due to perceived stigma. This may reflect socio-cultural differences in attitudes to dementia or coping with adversity, or the perceived risks or threats of having the diagnosis. Further exploration of this finding would be beneficial as the IDEAL cohort included small numbers from the most deprived areas.

There are some limitations to the study. The proportions of people with less common diagnoses and people from minority ethnic groups were representative of memory clinic attendance in Great Britain, but numerically the groups are small. Likewise, the number of people from more deprived areas are small. This may limit the generalizability of the results beyond the common presentations. No premorbid estimates of personality traits or psychological attributes were collected, so it is not possible to determine whether ratings were affected by dementia and/or degree of awareness. Information about what people with dementia had been told about their condition was not available; thus higher awareness of diagnosis may reflect better provision of information and services (and/or better recall). However, the criteria distinguishing the low awareness group from the rest of the cohort were likely to be applicable in most settings. There is no agreed gold standard for measuring awareness and this study did not set out to explore comprehensive awareness. However, structured enquiry allowing self-report of awareness concerning condition appears effective and an appropriate approach to person-centered assessments.

IDEAL relies on self-reported measures. Some authors have questioned the reliability of self-reports provided by people with dementia who have reduced awareness, and whether they can accurately appraise their circumstances [63]. Others confirmed the validity of self-report when measuring areas such as QoL which are essentially subjective [26, 51]. It is arguable that people who use a self-maintaining coping strategy in response to dementia might be both reluctant to acknowledge symptoms of dementia and eager to portray their QoL as unchanged. However, an overlap in the living well scores was seen between the awareness groups; some people in the low awareness group reported lower QoL, well-being or life-satisfaction than some in the high awareness group. We cannot be certain of the meaning of self-rated capability to live well in people who do not acknowledge significant symptoms of a life-changing illness. This may also be relevant for the self-reporting of mood, personality traits and positive psychological resources, also subject to social constructs and established psychological responses in healthy people as well as people with dementia. But these mechanisms may actually contribute to successfully maintaining subjective quality of life, well-being and satisfaction with life. The alternative method of using informant ratings is often used for observable behaviours and activities, but can be particularly problematic for subjective areas such as QoL [64]. Regarding living well, it would seem inappropriate to overlook the individual views of people living with dementia on this issue.

The current study investigated awareness of the condition of dementia - an under-researched area - using a validated checklist to screen for low awareness. The study comprised a large sample of people with dementia from a multi-center program, using self-report to categorize awareness which avoids possible biases from an informant or proxy. To our knowledge, this is the first study of awareness which investigates well-being and life satisfaction as well as QoL. Differences between the high and low awareness groups for the living well indices are clinically meaningful [27, 65] raising important issues about awareness of condition relevant to clinical care.

### Implications for future work and clinical use

This study suggests that awareness of condition varies in people living with dementia and is relevant to living well. Awareness of condition should be considered in clinical assessments for greater understanding of an individual’s position if interventions are aimed at optimizing living well. This may entail assessing awareness of condition around the time of disclosing a diagnosis, and awareness of diagnosis at subsequent post-diagnostic reviews.

Being able to acknowledge the diagnosis of dementia and its implications is central to discussions around treatment choices and advanced care planning e.g., discussing dementia medication, arranging power of attorney and wills and discussing wishes for end-of-life care. A recent review welcomes the advances in truthful disclosure of dementia diagnoses, but points to the ongoing need for tailored and phased communication [53]. For clinicians, being able to discuss the diagnosis with transparency allows open sharing of concerns when supporting someone with treatment planning*.* However, raising personal awareness of a dementia diagnosis may have negative implications for mood and the ability to live well for an individual with dementia. There is no indication here that it would always be helpful to enhance awareness of diagnosis, but it does suggest that it is important for clinicians to understand the level of awareness in order to tailor the information and support provided.

Causality cannot be determined in this study; longitudinal studies would be important to investigate the longer-term consequences of awareness. Awareness of condition may relate to social factors which should be explored further; equity of access to information is important. Further research looking more closely at disclosure of diagnosis will be valuable considering existing awareness and how to gauge individual preferences and readiness for life-changing news. A reliable method of assessing awareness in a clinical setting would be a helpful development. Meanwhile assumptions cannot be made about a person’s level of awareness, and information and support should continue to be handled sensitively and tailored to the individual.

## Conclusions

Awareness of dementia condition was assessed using self-report and among people with mild-to-moderate dementia, 90% showed some awareness of their condition. For those with either lower or higher awareness of condition there were contrasting findings regarding self-reported psychological health and ability to live well, favoring those with lower awareness. Psychosocial factors were important predictors of awareness. For people living with dementia, individual features including level of awareness should be considered when communicating diagnoses and offering support in person-centered service provision.

## Supplementary Information

**Additional file 1: Supplementary Table S1.** RADIX screening questions.

**Additional file 2: Supplementary Table S2.** Univariate logistic regression for awareness groups.

**Additional file 3: Supplementary Table S3.** Multivariate logistic regression for awareness groups; adjusted for age group, sex, dementia subtype and GDS-10 group.

**Additional file 4: Supplementary Fig. S1.** Bar chart showing dementia subtype and awareness groups.

**Additional file 5: Supplementary Figs. S2a, S2b, and S2c.** Boxplots showing living well indices and awareness groups.

## Data Availability

For the datasets supporting the conclusions of this study, IDEAL data were deposited with the UK data archive in April 2020 and will be available to access from April 2023. Details of how the data can be accessed after that date can be found here: http://reshare.ukdataservice.ac.uk/854293/

## Abbreviations

AD: Alzheimer’s disease
QoL: Quality of life
IDEAL: Improving the experience of Dementia and Enhancing Active Life
RADIX: Representations and Adjustment to Dementia Index
QoL-AD: Quality of Life in Alzheimer’s Disease Scale
ACE-III: Addenbrooke’s Cognitive Examination III
GDS-10: 10-item Geriatric Depression Scale
NPI-Q: Neuropsychiatric Inventory Questionnaire
