# Supplementary material for: Does awareness of condition help people with mild-to-moderate dementia to live well? Findings from the IDEAL programme
Source: BMC Geriatr. 2021 Sep 25;21:511. doi: 10.1186/s12877-021-02468-4 (PMC8467163; doi:10.1186/s12877-021-02468-4)
Supplement: Supplementary file 1 — Additional file 1: Supplementary Table S1. RADIX screening questions. [file 12877_2021_2468_MOESM1_ESM.docx]

**Supplementary Table S1. RADIX screening questions.**

| **RADIX Item** | **Response *** |
| --- | --- |
| Have you, a family member or doctor noticed that you have been having difficulty with concentration? | Yes/No |
| Have you, a family member or doctor noticed that you have been forgetful? | Yes/No |
| Have you, a family member or doctor noticed that you have been having difficulty remembering (e.g. recent events)? | Yes/No |
| Have you, a family member or doctor noticed that you have been having difficulty with thinking? | Yes/No |
| Have you, a family member or doctor noticed that you have been having difficulty with your ability to say what you want to say? | Yes/No |
| Have you, a family member or doctor noticed you have been having difficulty with your ability to manage your day-to-day activities? | Yes/No |
| Have you, a family member or doctor noticed you have been having difficulty with planning ahead? | Yes/No |
| Have you, a family member or doctor noticed you have been having difficulty with making decisions? | Yes/No |
| Are you different in some way to how you used to be? | Yes/No |

*Considered low awareness of condition if no questions were endorsed.
