# Supplementary material for: Does awareness of condition help people with mild-to-moderate dementia to live well? Findings from the IDEAL programme
Source: BMC Geriatr. 2021 Sep 25;21:511. doi: 10.1186/s12877-021-02468-4 (PMC8467163; doi:10.1186/s12877-021-02468-4)
Supplement: Supplementary file 2 — Additional file 2: Supplementary Table S2. Univariate logistic regression for awareness groups. [file 12877_2021_2468_MOESM2_ESM.docx]

**Supplementary Table S2**. **Univariate logistic regression for awareness groups.**

**S2.1. Factors associated with awareness.**

S2.1a. Demographic variables.

|  | **OR Low awareness (n=83) vs Rest of cohort (n=834)** | | | | **OR High awareness (n=103) vs Low awareness (n=83)** | | | |
| --- | --- | --- | --- | --- | --- | --- | --- | --- |
|  | **OR** | **(95% CI)** | **p-value** | **Missing cases (%)** | **OR** | **(95% CI)** | **p-value** | **Missing cases (%)** |
| **Age group** |  |  |  | 0 |  |  |  | 0 |
| <65y | .48 | (.20, 1.16) | .101 |  | 10.00 | (3.51, 28.53) | < .001 |  |
| 65-69y | .65 | (.32, 1.30) | .221 |  | 3.64 | (1.42, 9.33) | . 007 |  |
| 70-74y | .38 | (.18, .80) | .011 |  | 6.11 | (2.37, 15.78) | < .001 |  |
| 75-79y | .38 | (.20, .73) | .004 |  | 4.79 | (1.98, 11.63) | .001 |  |
| 80+y | Reference group |  |  |  | Reference group |  |  |  |
| **Sex** |  |  |  | 0 |  |  |  | 0 |
| Female | .91 | (.58, 1.45) | .697 |  | 1.13 | (.63, 2.03) | .684 |  |
| Male | Reference group |  |  |  | Reference group |  |  |  |
| **Time since diagnosis** |  |  |  | 68 (7.4) |  |  |  | 19 (10.2) |
| < 1yr | Reference group |  |  |  | Reference group |  |  |  |
| 1-2yr | .87 | (.49, 1.54) | .635 |  | 1.86 | (.92, 3.75) | .084 |  |
| 3yr and above | 1.27 | (.63, 2.58) | .501 |  | 1.52 | (.64, 3.62) | .345 |  |
| **Deprivation quintile** |  |  |  | 0 |  |  |  | 0 |
| Q1 (most deprived) | 1.94 | (.93, 4.03) | .077 |  | .44 | (.16, 1.23) | .117 |  |
| Q2 | 1.23 | (.60, 2.53) | .570 |  | .98 | (.40, 2.41) | .970 |  |
| Q3 | .95 | (.48, 1.85) | .873 |  | .93 | (.40, 2.18) | .870 |  |
| Q4 | 1.03 | (.54, 1.96) | .925 |  | .93 | (.42, 2.10) | .868 |  |
| Q5 (least deprived) | Reference group |  |  |  | Reference group |  |  |  |
| **Dementia subtype** |  |  |  | 0 |  |  |  | 0 |
| AD | Reference group |  |  |  | Reference group |  |  |  |
| VaD | 1.27 | (.65, 2.48) | .492 |  | 1.18 | (.51, 2.75) | .698 |  |
| Mixed | .78 | (.41, 1.47) | .441 |  | .96 | (.41, 2.24) | .916 |  |
| FTD | 2.54 | (1.05, 6.15) | .038 |  | .38 | (.09, 1.55) | .178 |  |
| PDD | .31 | (.04, 2.29) | .250 |  | 8.87 | (1.09, 71.90) | .041 |  |
| DLB | .27 | (.04, 2.03) | .205 |  | 3.55 | (.38, 32.86) | .265 |  |
| Other | .93 | (.21, 4.11) | .928 |  | 1.33 | (.21, 8.31) | .760 |  |
| **Education** |  |  |  | 19 (2.1) |  |  |  | 6 (3.2) |
| No qualification | 1.02 | (.56, 1.84) | .952 |  | .98 | (.46, 2.08) | .956 |  |
| School leaving certificate 16y | 1.05 | (.54, 2.04) | .890 |  | .74 | (.31, 1.79) | .507 |  |
| School leaving certificate 18y | Reference group |  |  |  | Reference group |  |  |  |
| University | .94 | (.48, 1.86) | .862 |  | 1.27 | (.55, 2.93) | .569 |  |

S2.1b. Cognitive variables.

|  | **OR Low awareness (n=83) vs Rest of cohort (n=834)** | | | | **OR High awareness (n=103) vs Low awareness (n=83)** | | | |
| --- | --- | --- | --- | --- | --- | --- | --- | --- |
|  | **OR** | **(95% CI)** | **p-value** | **Missing cases (%)** | **OR** | **(95% CI)** | **p-value** | **Missing cases (%)** |
| **ACE-III Total** | .97 | (.96, .99) | .004 | 71 (7.7) | 1.04 | (1.01, 1.06) | .004 | 18 (9.7) |
| **ACE-III attention** | .91 | (.84, .98) | .009 | 28 (3.1) | 1.10 | (1.00, 1.21) | .051 | 9 (4.8) |
| **ACE-III fluency** | .94 | (.87, 1.02) | .128 | 23 (2.5) | 1.07 | (.97, 1.18) | .156 | 8 (4.3) |
| **ACE-III language** | .94 | (.89, 1.00) | .054 | 51 (5.6) | 1.16 | (1.05, 1.28) | .004 | 14 (7.5) |
| **ACE-III memory** | .92 | (.87, .96) | .001 | 40 (4.4) | 1.13 | (1.06, 1.20) | <.001 | 14 (7.5) |
| **ACE-III visuospatial** | 1.00 | (.93, 1.07) | .904 | 35 (3.8) | 1.00 | (.92, 1.10) | .928 | 12 (6.5) |

S2.1c. Psychological variables.

|  | **OR Low awareness (n=83) vs Rest of cohort (n=834)** | | | | **OR High awareness (n=103) vs Low awareness (n=83)** | | | |
| --- | --- | --- | --- | --- | --- | --- | --- | --- |
|  | **OR** | **(95% CI)** | **p-value** | **Missing cases (%)** | **OR** | **(95% CI)** | **p-value** | **Missing cases (%)** |
| **GDS-10 group** |  |  |  | 20 (2.2) |  |  |  | 4 (2.2) |
| Depressed | .20 | (.09, .46) | <.001 |  | 6.93 | (2.90, 16.57) | <.001 |  |
| Not depressed | Reference group |  |  |  | Reference group |  |  |  |
| **Neuroticism** | .80 | (.74, .87) | <.001 | 35 (3.8) | 1.32 | (1.18, 1.46) | <.001 | 5 (2.7) |
| **Openness** | 1.04 | (.97, 1.12) | .287 | 43 (4.7) | .95 | (.87, 1.04) | .268 | 8 (4.3) |
| **Agreeable** | .95 | (.88, 1.03) | .200 | 31 (3.4) | 1.07 | (.97, 1.19) | .179 | 5 (2.7) |
| **Conscientious** | 1.17 | (1.08, 1.26) | <.001 | 35 (3.8) | .82 | (.73, .91) | <.001 | 6 (3.2) |
| **Extraversion** | 1.08 | (1.01, 1.15) | .018 | 31 (3.4) | .86 | (.79, .94) | .001 | 6 (3.2) |
| **Optimism** | 1.13 | (1.05, 1.21) | .001 | 34 (3.7) | .86 | (.79, .93) | < .001 | 10 (5.4) |
| **Self-efficacy** | 1.10 | (1.05, 1.16) | <.001 | 49 (5.3) | .87 | (.82, .93) | <.001 | 13 (7.0) |
| **Self-esteem** | 1.15 | (1.08, 1.22) | <.001 | 66 (7.2) | .84 | (.77, .92) | <.001 | 16 (9.7) |

S2.1d. Caregiver-rated variables.

|  | **OR Low awareness (n=67) vs Rest of cohort (n=688)** | | | | **OR High awareness (n=82) vs Low awareness (n=67)** | | | |
| --- | --- | --- | --- | --- | --- | --- | --- | --- |
|  | **OR** | **(95% CI)** | **p-value** | **Missing cases (%)** | **OR** | **(95% CI)** | **p-value** | **Missing cases (%)** |
| **FAQ-I** | 1.05 | (1.02, 1.09) | .002 | 58 (7.7) | .94 | (.90, .99) | .008 | 13 (8.7) |
| **NPI-Q total symptoms** | .97 | (.87, 1.08) | .557 | 38 (5.0) | 1.05 | (.91, 1.21) | .526 | 8 (5.4) |

**S2.2. Awareness and ability to live well**.

|  | **OR Low awareness (n=83) vs Rest of cohort (n=834)** | | | | **OR High awareness (n=103) vs Low awareness (n=83)** | | | |
| --- | --- | --- | --- | --- | --- | --- | --- | --- |
|  | **OR** | **(95% CI)** | **p-value** | **Missing cases (%)** | **OR** | **(95% CI)** | **p-value** | **Missing cases (%)** |
| **QoL-AD** | 1.16 | (1.11, 1.22) | <.001 | 67 (7.3) | .82 | (.77, .88) | <.001 | 15 (8.1) |
| **WHO-5** | 1.03 | (1.02, 1.05) | <.001 | 6 (0.7) | .96 | (.95, .98) | <.001 | 0 |
| **SwLS** | 1.11 | (1.06, 1.17) | <.001 | 12 (1.3) | .84 | (.79, .90) | <.001 | 1 (0.5) |

**S2.3. Awareness and caregiver stress.**

|  | **OR for Low awareness (n=67) vs Rest of cohort (n=688)** | | | | **OR for High awareness (n=82) vs Low awareness (n=67)** | | | |
| --- | --- | --- | --- | --- | --- | --- | --- | --- |
|  | **OR** | **(95% CI)** | **p-value** | **Missing cases (%)** | **OR** | **(95% CI)** | **p-value** | **Missing cases (%)** |
| **Caregiver RSS** | .99 | (.97, 1.02) | .660 | 46 (6.1) | 1.02 | (.98, 1.06) | .365 | 6 (4.0) |

OR Odds ratio; CI Confidence interval; AD Alzheimer’s disease; VaD vascular dementia; FTD frontotemporal dementia; PDD Parkinson’s disease dementia; DLB dementia with Lewy bodies; ACE-III Addenbrooke’s Cognitive Examination III; QoL-AD Quality of Life in Alzheimer’s Disease; SwLS Satisfaction with Life Scale; WHO-5 World Health Organization-Five Well-being Index; GDS-10 Geriatric Depression Scale-10; FAQ-I Functional Activities Questionnaire-Informant rated; NPI-Q Neuropsychiatric Inventory Questionnaire; RSS Relative Stress Scale.
