# Supplementary material for: Does awareness of condition help people with mild-to-moderate dementia to live well? Findings from the IDEAL programme
Source: BMC Geriatr. 2021 Sep 25;21:511. doi: 10.1186/s12877-021-02468-4 (PMC8467163; doi:10.1186/s12877-021-02468-4)
Supplement: Supplementary file 3 — Additional file 3: Supplementary Table S3. Multivariate logistic regression for awareness groups; adjusted for age group, sex, dementia subtype and GDS-10 group. [file 12877_2021_2468_MOESM3_ESM.docx]

**Supplementary Table S3. Multivariate logistic regression for awareness groups, adjusted for age group, sex, dementia subtype and GDS-10 group.**

**S3.1. Factors associated with awareness.**

S3.1a. Demographic variables

|  | **OR Low awareness (n=83) vs Rest of cohort (n=834)** | | | | **OR High awareness (n=103) vs Low awareness (n=83)** | | | |
| --- | --- | --- | --- | --- | --- | --- | --- | --- |
|  | **OR** | **(95% CI)** | **p-value** | **Missing cases (%)** | **OR** | **(95% CI)** | **p-value** | **Missing cases (%)** |
| **Time since diagnosis** |  |  |  | 87 (9.5) |  |  |  | 23 (12.4) |
| < 1yr | Reference group |  |  |  | Reference group |  |  |  |
| 1-2yr | .89 | (.49, 1.61) | .701 |  | 2.30 | (.94, 5.60) | .067 |  |
| 3yr and above | 1.35 | (.65, 2.83) | .423 |  | 2.11 | (.71, 6.26) | .179 |  |
| **Deprivation quintile** |  |  |  | 20 (2.2) |  |  |  | 4 (2.2) |
| Q1 (most deprived) | 2.57 | (1.18, 5.60) | .017 |  | .08 | (.02, .47) | .005 |  |
| Q2 | 1.61 | (.76, 3.42) | .213 |  | .56 | (.19, 1.68) | .300 |  |
| Q3 | 1.05 | (.52, 2.14) | .887 |  | .54 | (.18, 1.60) | .266 |  |
| Q4 | 1.26 | (.65, 2.45) | .501 |  | .71 | (.26, 1.97) | .516 |  |
| Q5 (least deprived) | Reference group |  |  |  | Reference group |  |  |  |
| **Education** |  |  |  | 36 (3.9) |  |  |  | 8 (4.3) |
| No qualification | 1.11 | (.60, 2.08) | .737 |  | 1.37 | (.53, 3.52) | .513 |  |
| School leaving certificate 16y | 1.20 | (.60, 2.40) | .601 |  | .37 | (.12, 1.14) | .083 |  |
| School leaving certificate 18y | Reference group |  |  |  | Reference group |  |  |  |
| University | .97 | (.48, 1.96) | .933 |  | 1.09 | (.39, 3.02) | .871 |  |

S3.1b. Cognitive variables

|  | **OR Low awareness (n=83) vs Rest of cohort (n=834)** | | | | **OR High awareness (n=103) vs Low awareness (n=83)** | | | | |
| --- | --- | --- | --- | --- | --- | --- | --- | --- | --- |
|  | **OR** | **(95% CI)** | **p-value** | **Missing cases (%)** | | **OR** | **(95% CI)** | **p-value** | **Missing cases (%)** |
| **ACE-III total** | .97 | (.95, .99) | .004 | 88 (9.6) | | 1.04 | (1.01, 1.07) | .022 | 21 (11.3) |
| **ACE-III attention** | .89 | (.82, .97) | .006 | 46 (5.0) | | 1.15 | (1.01, 1.31) | .032 | 12 (6.5) |
| **ACE-III fluency** | .92 | (.85, 1.00) | .049 | 41 (4.5) | | 1.13 | (.99, 1.28) | .062 | 11 (5.9) |
| **ACE-III language** | .94 | (.88, 1.00) | .048 | 69 (7.5) | | 1.13 | (1.01, 1.27) | .034 | 17 (9.1) |
| **ACE-III memory** | .93 | (.88, .98) | .005 | 57 (6.2) | | 1.11 | (1.02, 1.20) | .017 | 17 (9.1) |
| **ACE-III visuospatial** | .96 | (.89, 1.04) | .317 | 52 (5.7) | | 1.06 | (.94, 1.20) | .338 | 15 (8.1) |

S3.1c. Psychological variables

|  | **OR Low awareness (n=83) vs Rest of cohort (n=834)** | | | | **OR High awareness (n=103) vs Low awareness (n=83)** | | | |
| --- | --- | --- | --- | --- | --- | --- | --- | --- |
|  | **OR** | **(95% CI)** | **p-value** | **Missing cases (%)** | **OR** | **(95% CI)** | **p-value** | **Missing cases (%)** |
| **Neuroticism** | .83 | (.76, .90) | <.001 | 55 (6.0) | 1.22 | (1.07, 1.39) | .004 | 4 (2.2) |
| **Openness** | 1.01 | (.93, 1.09) | .876 | 63 (6.9) | 1.02 | (.91, 1.15) | .717 | 12 (6.5) |
| **Agreeable** | .94 | (.86, 1.02) | .154 | 50 (5.5) | 1.16 | (1.01, 1.33) | .034 | 9 (4.4) |
| **Conscientious** | 1.13 | (1.04, 1.23) | .004 | 54 (5.9) | .89 | (.78, 1.02) | .095 | 10 (5.4) |
| **Extraversion** | 1.05 | (.98, 1.12) | .138 | 51 (5.6) | .89 | (.80, .98) | .020 | 10 (5.4) |
| **Optimism** | 1.08 | (1.00, 1.17) | .048 | 51 (5.6) | .92 | (.82, 1.02) | .124 | 13 (7.0) |
| **Self-efficacy** | 1.09 | (1.03, 1.15) | .003 | 66 (7.2) | .89 | (.82, .96) | .002 | 17 (9.1) |
| **Self-esteem** | 1.11 | (1.04, 1.19) | .004 | 84 (9.2) | .90 | (.79, 1.02) | .087 | 20 (10.8) |

S3.1d. Caregiver rated variables

|  | **OR Low awareness (n=67) vs Rest of cohort (n=688)** | | | | **OR High awareness (n=82) vs Low awareness (n=67)** | | | |
| --- | --- | --- | --- | --- | --- | --- | --- | --- |
|  | **OR** | **(95% CI)** | **p-value** | **Missing cases (%)** | **OR** | **(95% CI)** | **p-value** | **Missing cases (%)** |
| **FAQ-I** | 1.06 | (1.02, 1.10) | .001 | 69 (9.1) | .92 | (.87, .98) | .005 | 14 (9.4) |
| **NPI-Q total symptoms** | .99 | (.88, 1.10) | .827 | 48 (6.4) | .99 | (.83, 1.19) | .927 | 9 (6.0) |

**S3.2. Awareness and the ability to live well.**

|  | **OR Low awareness (n=83) vs Rest of cohort (n=834)** | | | | **OR High awareness (n=103) vs Low awareness (n=83)** | | | |
| --- | --- | --- | --- | --- | --- | --- | --- | --- |
|  | **OR** | **(95% CI)** | **p-value** | **Missing cases (%)** | **OR** | **(95% CI)** | **p-value** | **Missing cases (%)** |
| **QoL-AD** | 1.15 | (1.09, 1.22) | <.001 | 84 (9.2) | .84 | (.76, .92) | <.001 | 19 (10.2) |
| **WHO-5** | 1.02 | (1.01, 1.04) | .007 | 26 (2.8) | .98 | (.96, 1.00) | .048 | 4 (2.2) |
| **SwLS** | 1.08 | (1.02, 1.14) | .005 | 31 (3.4) | .85 | (.78, .93) | <.001 | 4 (2.2) |

**S3.3. Awareness and caregiver stress.**

|  | **OR Low awareness (n=67) vs Rest of cohort (n=688)** | | | | **OR High awareness (n=82) vs Low awareness (n=67)** | | | |
| --- | --- | --- | --- | --- | --- | --- | --- | --- |
|  | **OR** | **(95% CI)** | **p-value** | **Missing cases (%)** | **OR** | **(95% CI)** | **p-value** | **Missing cases (%)** |
| **Caregiver RSS** | 1.00 | (.97, 1.03) | .947 | 56 (7.4) | 1.01 | (.96, 1.06) | .776 | 7 (4.7) |

OR Odds Ratio; CI Confidence interval; AD Alzheimer’s disease; VaD vascular dementia; FTD frontotemporal dementia; PDD Parkinson’s disease dementia; DLB dementia with Lewy bodies; ACE-III Addenbrooke’s Cognitive Examination III; QoL-AD Quality of Life in Alzheimer’s Disease; SwLS Satisfaction with Life Scale; WHO-5 World Health Organization-Five Well-being Index; GDS-10 Geriatric Depression Scale-10; FAQ-I Functional Activities Questionnaire-Informant rated; NPI-Q Neuropsychiatric Inventory Questionnaire; RSS Relative Stress Scale.
