# Supplementary material for: Does awareness of condition help people with mild-to-moderate dementia to live well? Findings from the IDEAL programme
Source: BMC Geriatr. 2021 Sep 25;21:511. doi: 10.1186/s12877-021-02468-4 (PMC8467163; doi:10.1186/s12877-021-02468-4)
Supplement: Supplementary file 4 — Additional file 4: Supplementary Fig. S1. Bar chart showing dementia subtype and awareness groups. [file 12877_2021_2468_MOESM4_ESM.docx]

**Supplementary Figure S1. Dementia subtype and awareness group:** **Bar chart showing breakdown of each awareness group by dementia subtype.**

AD Alzheimer’s disease (n=508); VaD vascular dementia (n=105); Mixed (n=177); FTD frontotemporal dementia (n=34); PDD Parkinson’s disease dementia (n=33); DLB dementia with Lewy bodies (n=37); Other (n=23).
