# Supplementary material for: Does awareness of condition help people with mild-to-moderate dementia to live well? Findings from the IDEAL programme
Source: BMC Geriatr. 2021 Sep 25;21:511. doi: 10.1186/s12877-021-02468-4 (PMC8467163; doi:10.1186/s12877-021-02468-4)
Supplement: Supplementary file 5 — Additional file 5: Supplementary Figs. S2a, S2b, and S2c. Boxplots showing living well indices and awareness groups. [file 12877_2021_2468_MOESM5_ESM.docx]

**Supplementary Figures S2a, S2b, S2c. Boxplots showing living well indices and awareness groups.**

S2a. QoL-AD total score and awareness group

S2b. WHO-5 percent score and awareness group

S2c. SwLS total score and awareness group

QoL-AD Quality of Life-Alzheimer’s Disease scale; WHO-5 World Health Organization-Five Well-being Index; SwLS Satisfaction with Life Scale.
